# Membrane progesterone receptor beta (mPRβ/Paqr8) promotes progesterone-dependent neurite outgrowth in PC12 neuronal cells via non-G protein-coupled receptor (GPCR) signaling

**DOI:** 10.1038/s41598-017-05423-9

**Published:** 2017-07-12

**Authors:** Mayu Kasubuchi, Keita Watanabe, Kanako Hirano, Daisuke Inoue, Xuan Li, Kazuya Terasawa, Morichika Konishi, Nobuyuki Itoh, Ikuo Kimura

**Affiliations:** 1 Department of Applied Biological Science, Graduate School of Agriculture, Tokyo University of Agriculture and Technology, Fuchu-shi Tokyo, 183-8509 Japan; 2 Department of Genetic Biochemistry, Kyoto University Graduate School of Pharmaceutical Science, Sakyo Kyoto, 606-8501 Japan; 3 Center for Innovation in Immunoregulative Technology and Therapeutics, Kyoto University Graduate School of Medicine, Sakyo Kyoto, 606-8501 Japan; 4 Department of Microbial Chemistry, Kobe Pharmaceutical University, Higashinada Kobe, 658-8558 Japan

## Abstract

Recently, sex steroid membrane receptors garnered world-wide attention because they may be related to sex hormone-mediated unknown rapid non-genomic action that cannot be currently explained by their genomic action via nuclear receptors. Progesterone affects cell proliferation and survival via non-genomic effects. In this process, membrane progesterone receptors (mPRα, mPRβ, mPRγ, mPRδ, and mPRε) were identified as putative G protein-coupled receptors (GPCRs) for progesterone. However, the structure, intracellular signaling, and physiological functions of these progesterone receptors are still unclear. Here, we identify a molecular mechanism by which progesterone promotes neurite outgrowth through mPRβ (Paqr8) activation. Mouse *mPRβ* mRNA was specifically expressed in the central nervous system. It has an incomplete GPCR topology, presenting 6 transmembrane domains and did not exhibit typical GPCR signaling. Progesterone-dependent neurite outgrowth was exhibited by the promotion of ERK phosphorylation via mPRβ, but not via other progesterone receptors such as progesterone membrane receptor 1 (PGRMC-1) and nuclear progesterone receptor in nerve growth factor-induced neuronal PC12 cells. These findings provide new insights of regarding the non-genomic action of progesterone in the central nervous system.

## Introduction

Steroid hormones such as corticosterone, progesterone, testosterone, and estrogen are known to exhibit their physiological effects via their specific nuclear receptors^1^. Steroid hormones regulate gene transcription through nuclear receptors, which act as ligand-dependent transcription factors. These effects are known as “genomic” actions of steroid hormones, which generally take few hours to days to fully manifest. However, in various tissues, including the central nervous system (CNS), steroid hormones present a rapid action on the targeted cells within minutes. These “non-genomic” actions can be partially explained by membrane transport via nuclear receptors^2, 3^. However, other “non-genomic” actions are nuclear receptor-independent responses caused by insensitivity to the receptor antagonist and have been observed in knockout mice^4^. This suggests the possible involvement of unidentified receptors in the rapid non-genomic actions of steroid hormones^5^. The putative receptors for these actions have not yet been identified.

In the late 1990s, membrane progesterone receptors (mPRs), putative G protein-coupled receptors (GPCRs), and GPR30, one of the typical GPCRs, were identified as the membrane receptors for progesterone and estrogen, respectively^6–8^. Meanwhile, progesterone receptor membrane component-1 (PGRMC-1) and PGRMC-2, two single transmembrane proteins, were also identified as the putative membrane receptors for progesterone^9–11^. In contrast to the nuclear receptors, these membrane receptors mediate the rapid non-genomic effects of steroid hormones, such as the activation of MAPK signaling and intracellular Ca^2+^ increase^4, 7, 12–14^.

mPRβ/Paqr8 belongs to the progestin and AdipoQ receptor (PAQR) family, which contains 4 adiponectin-like receptors (class I receptors), 5 unique mPR members mPRα, mPRβ, mPRγ, mPRδ, and mPRε, class II receptors), and 2 hemolysin receptor like receptors^15–17^. mPRs can sense and respond to progesterone with EC50 values that are physiologically relevant^18, 19^. Thomas *et al*. reported that mPRα and mPRβ are typical GPCRs because progesterone activates a pertussis toxin-sensitive inhibitory G protein (G(i)) to down-regulate membrane-bound adenylyl cyclase (cAMP) activity in mPRα-transfected cells^20^. On the contrary, Smith *et al*. reported that mPRα and mPRγ are not GPCRs because in heterologous expression of human mPRα and mPRγ, their progesterone-dependent signaling in yeast does not require heterotrimeric G proteins^19^. In addition, mPRs belong to the Paqr family. AdipoR1 (Paqr1) and AdipoR2 (Paqr2) are not GPCRs and possess 7 transmembrane domains, in contrast to GPCRs in the membrane^21^. Thus, the topology of mPRs remains controversial. mPRα and mPRβ are abundantly expressed in the mouse brain, including the hypothalamus and midbrain. Their expression may be associated with the functional effects of progesterone in hormone-primed mice for lordosis^22, 23^ and with neuroprotective effects of progesterone in neurological diseases such as ischemic stroke, traumatic brain injury, and subarachnoid hemorrhage^4, 24^.

The detailed molecular mechanism underlying progesterone-dependent mPRβ activation in neural cells is still unclear. In this study, using a heterologous expression system and neural cell lines, we identified the intracellular signaling pathway underlying mPRβ activation and its physiological functions.

## Results

### mPRβ is specifically expressed in the CNS

**Figure 1:**
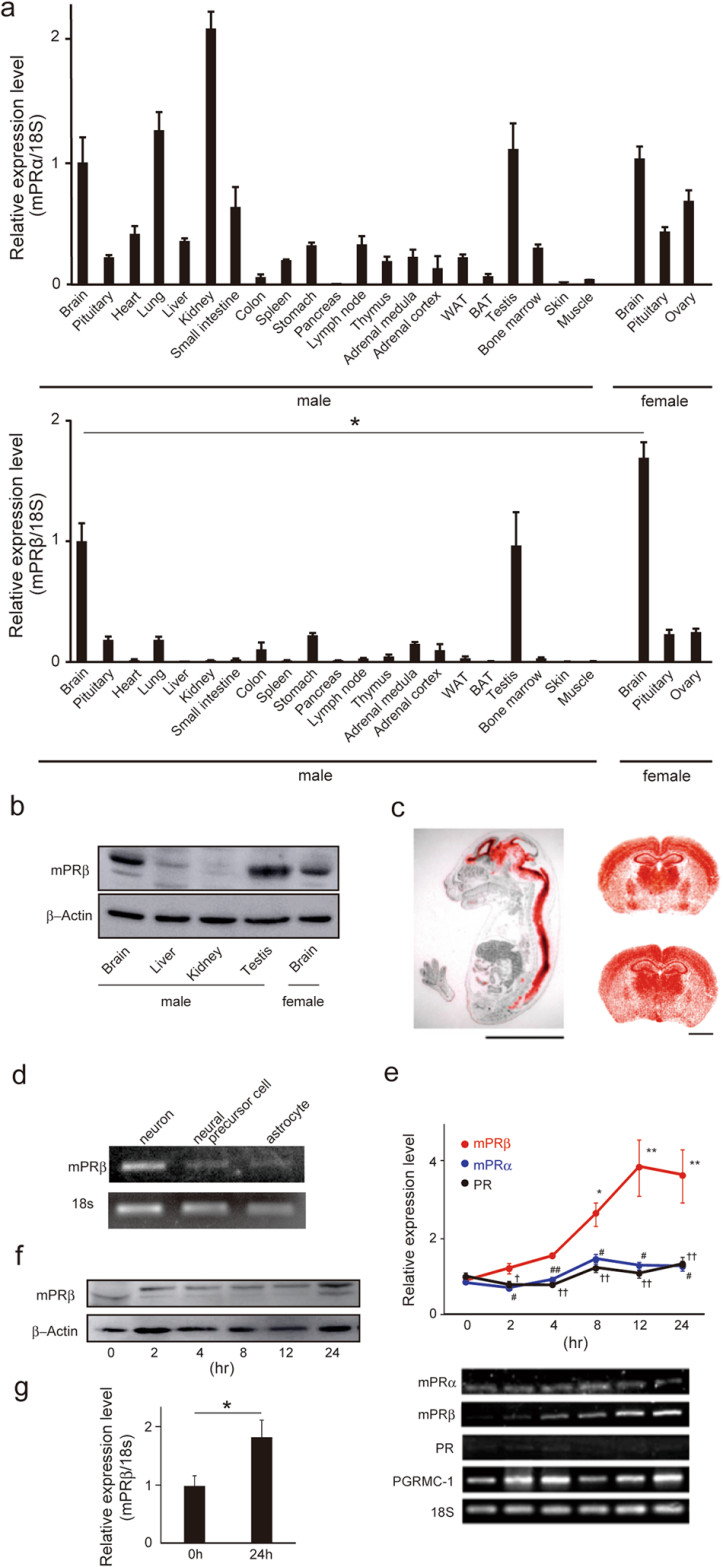
*mPRβ* is specifically expressed in the brain. (**a**) Expression of mPRα and mPRβ mRNA in mouse tissues (Post-natal day 49: P49) measured by quantitative RT-PCR (n = 3). WAT: White adipose tissue (epididymal adipose tissue), BAT: Brown adipose tissue. Control: *18S* mRNA expression. Statistical analysis was performed by using Student’s t-test. (**b**) Expression of mPRβ protein in mouse tissues (Post-natal day 49: P49) measured by western blotting. β-actin protein expression was used as an internal control. (**c**) Localization of mPRβ mRNA in mouse embryos (E15.5, sagittal sections, Scale bar = 5 mm) and mouse brain (upper: male, lower: female, P49, coronal sections, Scale bar = 2 mm). They were examined by *in situ* hybridization with a ^35^S-labeled antisense mouse mPRβ RNA probe. Red grains superimposed on a hematoxylin-eosin stain indicate the localization of mPRβ mRNA. (**d**) mPRβ cDNA (about 600 base pairs) was detected in neurons, neural precursor cells, and astrocytes by 1.5% agarose gel electrophoresis followed by staining with ethidium bromide. *18S* mRNA expression was used as an internal control. (**e**) The expression of the progesterone receptor was examined by quantitative RT-PCR in NGF-induced neuronal PC12 cells. (n = 3–6). *p < 0.05, and **p < 0.01, compared with 0 h mPRβ; ^#^ *p* < 0.05, and ^##^ *p* < 0.01, compared with mPRβ; ^†^ *p* < 0.05, and ^††^ *p* < 0.01, compared with mPRβ (Tukey-Kramer). PR: Progesterone Receptor. (**f**) mPRb protein expression in NGF-induced neuronal PC12 cells. β-actin protein expression was used as an internal control. (**g**) Expression of mPRβ mRNA in NGF-induced neuronal SH-SY5Y cells. Statistical analysis was performed by using Student’s t-test. Results are presented as means ± S.E.M. *p < 0.05.

We first examined mPRα and mPRβ expression in mice. The expression of *mPRα* and *mPRβ* mRNA in mice tissues on postnatal day 49 (P49), during sexual maturation, was examined by real-time quantitative RT-PCR. *mPRα* mRNA was detected in various tissues, including the brain, lung, kidney, and testis, whereas *mPRβ* mRNA was specifically detected in the brain both in males and females (Fig. 1a). The *mPRβ* mRNA expression was significantly higher in the female brain than in the male brain (Fig. 1a). The mPRβ protein was also detected in the brain (Fig. 1b). The expression of *mPRβ* mRNA in mouse embryos (Embryonic day 18.5) and in the brain (P49) was also examined by *in situ* hybridization. *mPRβ* mRNA was abundantly expressed in the developing CNS such as the brain and spinal cord. In the adult brain (P49), *mPRβ* expression was abundant and widespread, particularly in the cerebral cortex, hippocampus, and thalamus in both males and females (Fig. 1c). In primary cultured cerebral cortex neural cells, *mPRβ* mRNA was detected in neurons, but not neural precursor cells and astrocytes (Fig. 1d). *mPRβ* mRNA was drastically increased during NGF-induced neurogenesis in PC12, a rat adrenal pheochromocytoma cell line, whereas the expression of other progesterone receptors such as mPRα, Progesterone Receptor (PR), and PGRMC-1 did not exhibit the same expression profile (Fig. 1e). mPRβ protein was also drastically increased during neurogenesis in PC12 cells (Fig. 1f). Additionally, *mPRβ* mRNA was significantly increased in the NGF-induced neuronal human neuroblastoma cell lines SH-SY5Y as well (Fig. 1g). Thus, mPRβ is expressed specifically in the CNS, especially in mature neurons.

### Progesterone promotes neurite outgrowth via mPRβ in NGF-induced neuronal PC12 cells

**Figure 2:**
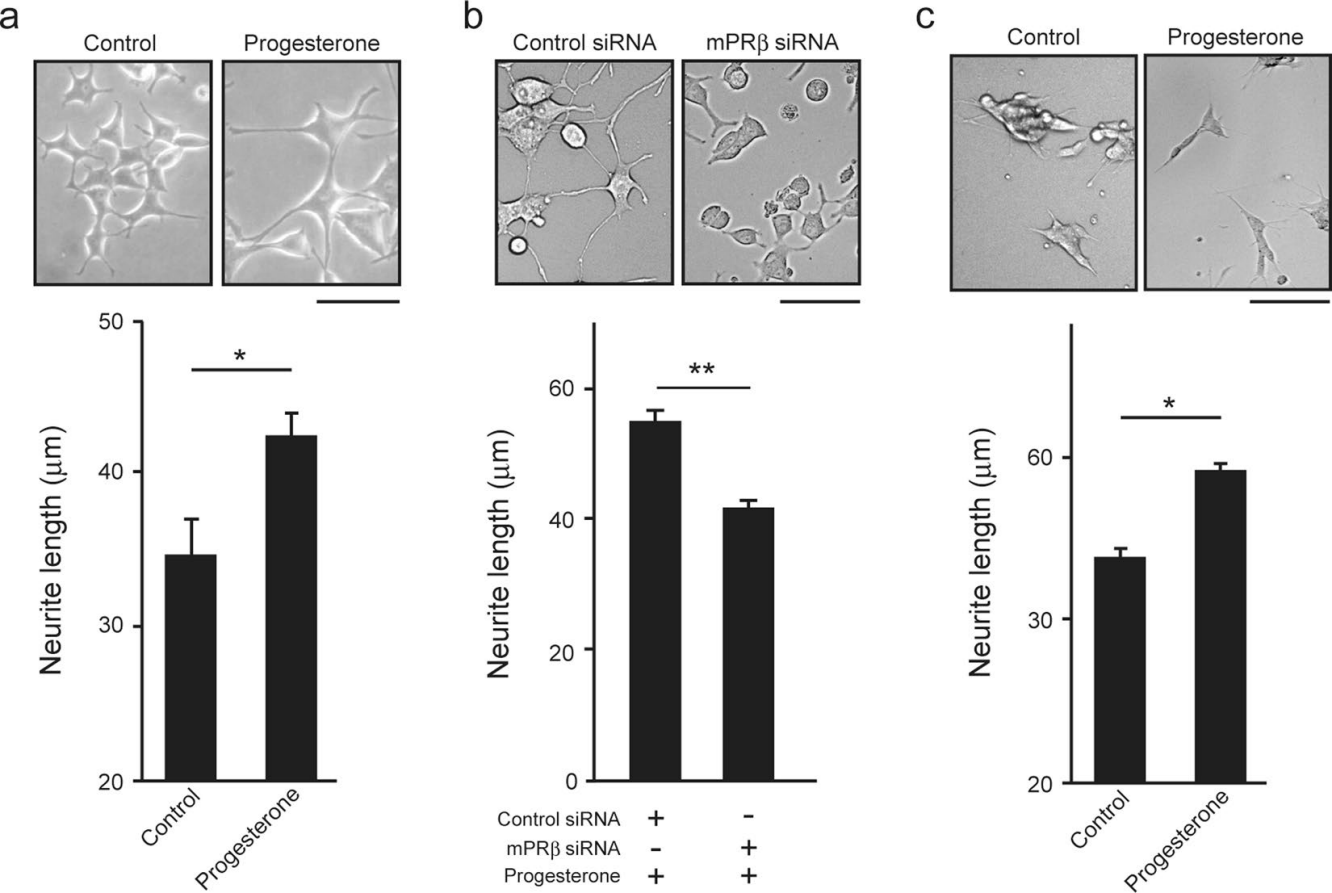
Effects of progesterone on neurite outgrowth via mPRβ in NGF-induced neuronal PC12 cells. (**a**) Effects of progesterone on neurite outgrowth. After 24 h in culture, PC12 cells were treated with NGF (50 ng/mL) or co-stimulated with NGF and progesterone (10 μM) for 3 days. (n = 3). Scale bar = 100 μm. (**b**) After being treated with Control siRNA or mPRβ siRNA, PC12 cells were cultured for 3 days in DMEM containing 1% FBS, NGF (50 ng/mL) and progesterone (10 μM) (n = 3). (**c**) Effects of progesterone on neurite outgrowth. After 24 h in culture, SH-SY5Y cells were treated with NGF (50 ng/mL) or co-stimulated with NGF and progesterone (10 μM) for 12 h. (n = 4–8). Scale bar = 100 μm. The graph reports the average length of neurites. Results are presented as means ± S.E.M. *p < 0.05, **p < 0.01. Statistical analysis was performed by using Student’s t-test.

We next examined the effects of mPRβ on neurite outgrowth in PC12 cells. PC12 cells were cultured in the presence of NGF (50 ng/mL) and treated with or without progesterone (10 μM) for 3 days. Progesterone-treated cultures presented longer neurites than those in control cultures (Fig. 2a). To elucidate whether this progesterone-dependent neurite outgrowth^25^ is related to mPRβ, we silenced mPRβ using RNAi. The real-time quantitative RT-PCR experiment revealed that mPRβ siRNA, but not control siRNA, suppressed *mPRβ* mRNA expression (Supp Fig. 1a) and significantly suppressed the promotion of progesterone-dependent neurite outgrowth in NGF-induced differentiated PC12 cells (Fig. 2b). As observed in PC12 cells, progesterone significantly promoted neurite outgrowth in NGF-induced differentiated SH-SY5Y cells^26^ (Fig. 2c). Thus, mPRβ mediates the progesterone-dependent neurite outgrowth.

### mPRβ stimulation by progesterone promotes ERK phosphorylation via non-GPCR signaling

**Figure 3:**
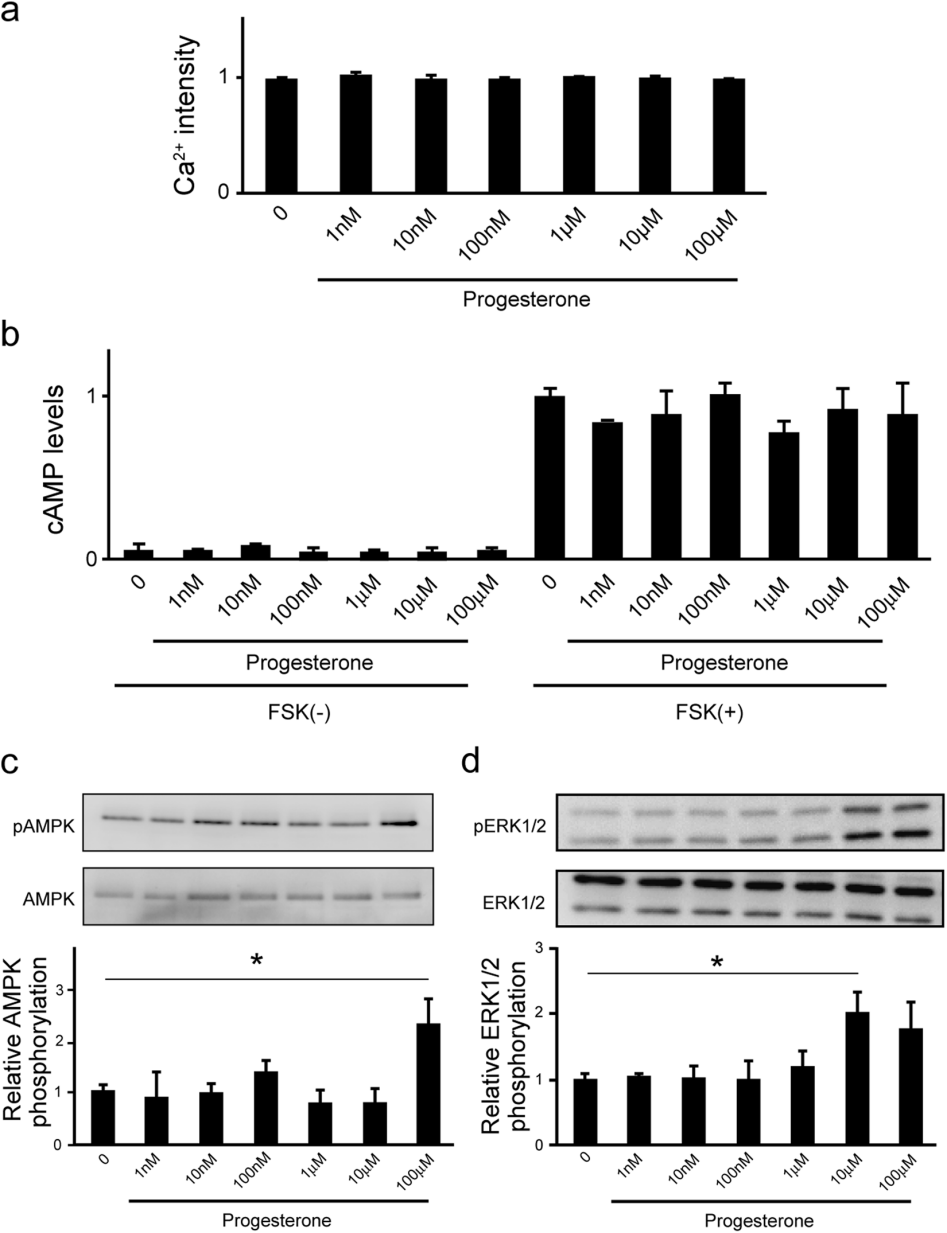
Progesterone promotes ERK phosphorylation via non-GPCR signaling in NGF-induced neuronal PC12 cells. (**a**) Mobilization of [Ca^2+^]_i_ induced by progesterone was monitored in PC12 cells, and data are presented as relative Ca^2+^ intensity. After 2 h in culture, cells were treated with NGF (50 ng/mL) and further cultured in DMEM containing 1% FBS for 24 h. (n = 3). (**b**) cAMP levels in response to progesterone treatment in PC12 cells. After 24 h in culture, NGF-induced PC12 cells pre-cultured with IBMX for 30 min were cultured in the presence of progesterone for 10 min. The cAMP levels in the cells were determined by using a cAMP EIA kit. (n = 3). (**c**) Effects of progesterone on AMPK phosphorylation in PC12 cells. After 24 h of culture, NGF-induced neuronal PC12 cells were further cultured for 3 h in serum-free DMEM. The cells were cultured in the presence of progesterone for 10 min. AMPK and its phosphorylated form were detected by western blotting with specific antibodies. (n = 5) (**d**) Agonistic effects of progesterone on ERK1/2 phosphorylation in PC12 cells. After 24 h of culture, NGF-induced neuronal PC12 cells were further cultured for 3 h in serum-free DMEM. The cells were cultured in the presence of progesterone for 10 min. ERK1/2 and its phosphorylated form were detected by western blotting with specific antibodies. (n = 3). Statistical analysis was performed by using one-way analysis of variance followed by Tukey-Kramer’s post hoc test, compared with control. FSK: Forskolin. Results are presented as means ± S.E.M. of independent wells.

We further examined whether progesterone activates GPCR signaling^27, 28^ such as Ca^2+^, cAMP, and ERK phosphorylation in NGF-induced neuronal PC12 cells. However, progesterone (1 nM–100 μM) did not affect Gq-coupled GPCR mediated intracellular calcium mobilization in NGF-induced neuronal PC12 cells (Fig. 3a). Progesterone (1 nM–100 μM) did not affect intracellular cAMP concentration, indicating that Gs and Gi/o coupled GPCR were not stimulated by progesterone in NGF-induced neuronal PC12 cells (Fig. 3b). On the other hand, progesterone (10 μM and 100 μM) promoted the phosphorylation of ERK in NGF-induced neuronal PC12 cells (Fig. 3c,d).

**Figure 4:**
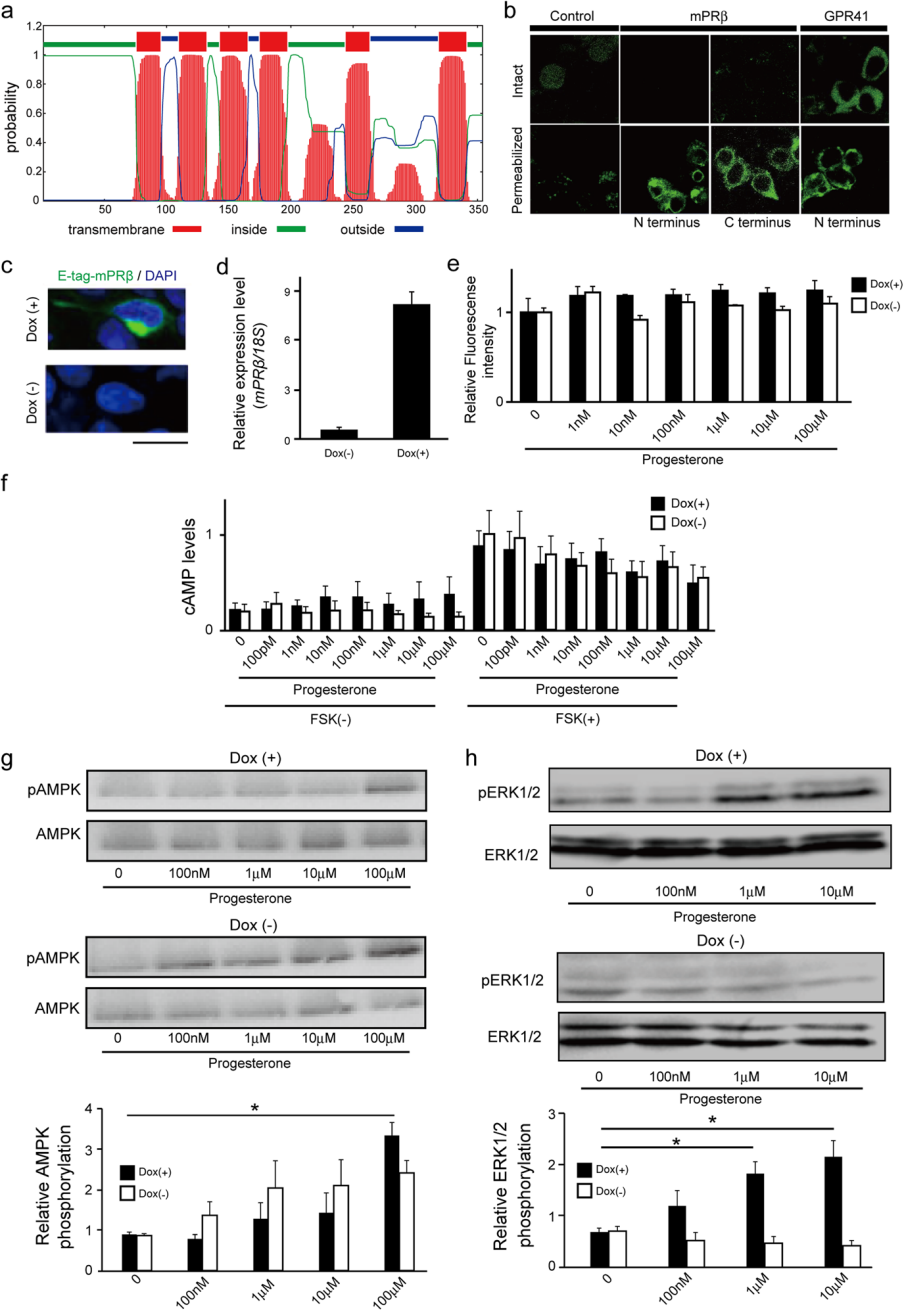
mPRβ stimulation by progesterone promotes ERK phosphorylation via non-GPCR signaling. (**a**) Prediction of transmembrane regions of mPRβ by using TMHMM 2.0 program. (**b**) Localization of mPRβ or GPR41 with epitope tags at either end. (**c**) The expression of mPRβ from the Flp-In locus was induced by treatment with 10 μg/mL doxycycline. After 24 h in culture, Flp in mPRβ T-Rex HEK293 cells were examined by immunochemistry with an anti-E-tag antibody. Green signals indicate mPRβ expression and blue signals indicate cell nuclei counter-stained with DAPI. (Scale bar = 20 μm). (**d**) Expression of *mPRβ* mRNA in Flp in mPRβ T-Rex HEK293 cells. Expression of mPRβ was measured using quantitative RT-PCR. *18S* mRNA expression was used as an internal control. (n = 3). (**e**) Mobilization of [Ca^2+^]_i_ induced by progesterone was monitored in Flp in mPRβ T-Rex HEK293 cells, and data are presented as relative Ca^2+^ intensity. After 2 h in culture, cells were treated with or without 10 μg/mL doxycycline. (n = 3). (**f**) cAMP levels in response to progesterone treatment in Flp in mPRβ T-Rex HEK293 cells. After 24 h in culture, cells were treated with or without 10 μg/mL doxycycline and further cultured for 24 h. Cells pre-cultured with IBMX for 30 min were cultured in the presence of progesterone for 10 min. The cAMP levels in the cells were determined by using a cAMP EIA kit. (n = 4). (**g**) Effects of progesterone on AMPK phosphorylation in Flp in mPRβ T-Rex HEK293 cells. After 24 h in culture with or without doxycycline (10 μg/mL), cells were further cultured for 24 h in serum-free DMEM. The cells were cultured in the presence of progesterone for 10 min. (n = 5) (**h**) Effects of progesterone on ERK1/2 phosphorylation in Flp in mPRβ T-Rex HEK293 cells. After 24 h in culture with or without doxycycline (10 μg/mL), cells were further cultured for 24 h in serum-free DMEM. The cells were cultured in the presence of progesterone for 10 min. Dox: Doxycycline. (n = 3).

In addition, using TMHMM sever, prediction of membrane helices in mPRβ from its amino acid sequence, showed that mPRβ presents incomplete 7 transmembrane domains and instead presents 6 transmembrane domains with cytoplasmic N- and C-termini (Fig. 4a). Hence, we examined mPRβ topology by immunohistochemistry using an epitope tag. mPRβ with the N- or C-terminus epitope tag was detected at the cell surface only in permeabilized cells, whereas a typical GPCR, GPR41 with the N-terminus epitope tag, was detected at the cell surface in non-permeabilized cells (Fig. 4b). Thus, mPRβ presents an incomplete GPCR topology. Furthermore, we also characterized mPRβ using a heterologous expression system in HEK293 cells^29^ (Fig. 4c,d). As in PC12 cells, mPRβ stimulation by progesterone did not induce Ca^2+^ increase, intracellular cAMP mobilization (Fig. 4e,f). Moreover, stimulation by progesterone promoted AMPK phosphorylation both in mPRβ-expressing and non-expressing HEK293 cells, but it promoted ERK phosphorylation in doxycycline-induced mPRβ overexpressing HEK293 cells (Fig. 4g,h). Thus, we confirmed that mPRβ is not a GPCR.

### Progesterone-stimulated mPRβ promotes neurite outgrowth via the PI3K-Rac1-MAPK cascade in NGF-induced neuronal PC12 cells

**Figure 5:**
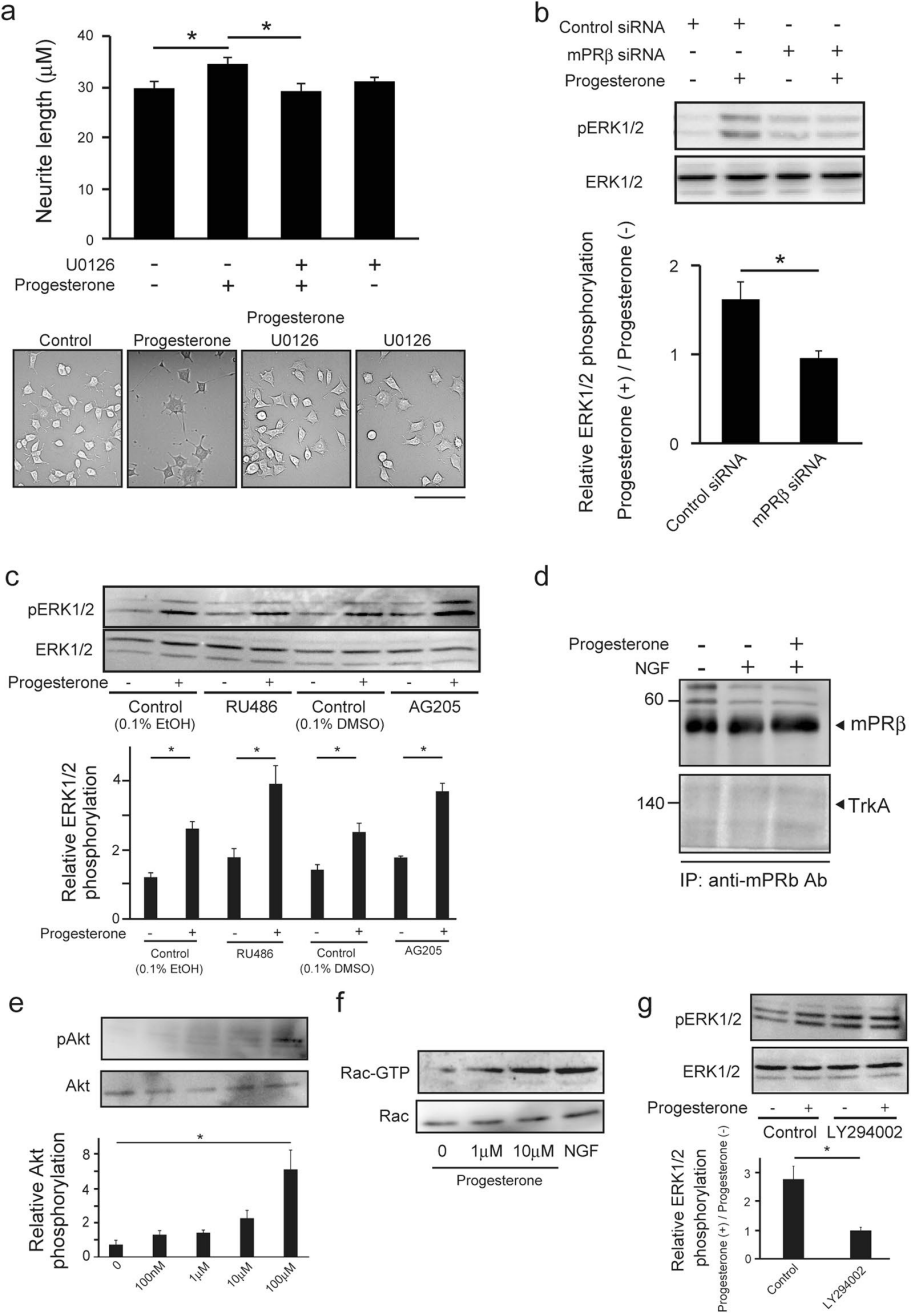
Inhibition of progesterone-mPRβ-MAPK signaling in PC12 cells suppresses neurite outgrowth. (**a**) Inhibitory effects of MEK inhibitor (U0126) on progesterone-induced neurite outgrowth in NGF-induced neuronal PC12 cells. After 24 h in culture, cells were further cultured in DMEM containing NGF (50 ng/mL), 1% FBS, with or without U0126 (10 μM) and progesterone (10 μM) for 3 days. Scale bar = 200 μm. (n = 3–5). Statistical analysis was performed by using one-way analysis of variance followed by Tukey-Kramer’s post hoc test. (**b**) Inhibitory effects of mPRβ siRNA on the phosphorylation of ERK1/2 in NGF-induced neuronal PC12 cells. After being treated with Control siRNA or mPRβ siRNA, cells were cultured for 3 days in DMEM containing 1% FBS, NGF (50 ng/mL) and with or without progesterone (10 μM). ERK1/2 and phosphorylated ERK1/2 in cells were detected by western blotting with specific antibodies. (n = 3). Statistical analysis was performed by using Student’s t-test. (**c**) Effects of progesterone (10 μM) on the phosphorylation of ERK1/2 in the presence or absence of RU486 (10 μM) and AG205 (10 μM) in PC12 cells. After 24 h in culture, cells were cultured in DMEM containing NGF (50 ng/mL) and 1% FBS. Cells were further cultured for 3 h in serum-free DMEM. After precultured with RU486 (10 μM) or AG205 (10 μM) for 30 min, cells were cultured in the presence or absence of progesterone (10 μM) for 10 min. (n = 5) Statistical analysis was performed by using one-way analysis of variance followed by Tukey-Kramer’s post hoc test. (**d**) Cells were left untreated or treated for 5 min with the progesterone (10 μM) or NGF (100 ng/ml). Lysate proteins were immune-precipitated with anti-mPRβ antibodies. The anti-mPRβ antibodies was used to detect mPRβ and anti-TrkA antibodies was used to detect TrkA. (**e**) Effects of progesterone on Akt phosphorylation in PC12 cells. After 24 h of culture, NGF-induced neuronal PC12 cells were further cultured for 3 h in serum-free DMEM. The cells were cultured in the presence of progesterone for 10 min. AKT and its phosphorylated form were detected by western blotting with specific antibodies. (n = 6) Statistical analysis was performed by using one-way analysis of variance followed by Tukey-Kramer’s post hoc test. (**f**) Effects of progesterone on Rac1 activation in PC12 cells. After 24 h of culture, NGF-induced neuronal PC12 cells were further cultured for 3 h in serum-free DMEM. The cells were cultured in the presence of progesterone or NGF (50 ng/ml) for 10 min. Rac activation was analyzed by pull-down assay. Active (Rac-GTP) or total Rac (Rac1) was detected by Western blot. (**g**) Effects of progesterone (10 μM) on the phosphorylation of ERK1/2 in the presence or absence of LY294002 (10 μM) in PC12 cells. After 24 h in culture, cells were cultured in DMEM containing NGF (50 ng/mL) and 1% FBS. Cells were further cultured for 3 h in serum-free DMEM. After precultured with LY294002 for 30 min, cells were cultured in the presence or absence of progesterone (10 μM) for 10 min. (n = 5). Statistical analysis was performed by using Student’s t-test. Results are presented as means ± S.E.M. *p < 0.05.

As described above, progesterone-stimulated mPRβ promoted ERK phosphorylation in NGF-induced neuronal PC12 cells. Therefore, we examined the role of the MAPK pathway in the effect of progesterone-mPRβ on neurite outgrowth. The MEK inhibitor, U0126, significantly inhibited the increase in neurite outgrowth induced by progesterone in NGF-induced neuronal PC12 cells (Fig. 5a). Moreover, mPRβ siRNA significantly suppressed the progesterone-stimulated ERK phosphorylation (Fig. 5b, Supp Fig. 1b), whereas PR antagonist, RU486^30^, and PGRMC-1 inhibitor, AG205, had no effect (Fig. 5c). Thus, progesterone promotes neurite outgrowth in NGF-induced neuronal PC12 cells through activation of MAPK cascade via mPRβ, but not via other progesterone receptors such as PR and PGRMC-1. To further clarify the effects of progesterone-mPRb signaling on neurite outgrowth, we examined whether this cross-talk between NGF and P4 for the promotion of neurite outgrowth is dependent on the association between TrkA and mPRb such as the previously revealed dependence on the association between TrkA and androgen receptor^2^. The results of immunoprecipitation did not indicate direct binding between TrkA and mPRb (Fig. 5d). Moreover, we examined the relationship between progesterone and PI3K cascade, known as the intracellular pathway for neurite outgrowth, as well as the MAPK cascade. Progesterone promotes Akt phosphorylation in the PI3K cascade (Fig. 5e) and activates Rac1 associated NGF-induced neurite outgrowth via the PI3K and MAPK cascades^31, 32^ (Fig. 5f). Additionally, inhibition of the PI3K cascade by LY294003, PI3K inhibitor, suppressed progesterone stimulated ERK phosphorylation (Fig. 5g). Thus, progesterone may promote neurite outgrowth via the mPRb-PI3K-Rac1-MAPK cascade.

## Discussion

The high expression of *mPRβ* in the CNS indicated that mPRβ may play an important role in the CNS-related progesterone effects. Real-time quantitative RT-PCR showed that mPRβ is specifically expressed in the brain in both males and females, while mPRα is ubiquitously expressed. Among the mPRs, mPRβ is specifically expressed in the CNS from the developing to the adult stage^33, 34^. Furthermore, mPRβ expression, but not that of other progesterone receptors such as PGRMC1 and PR is dramatically increased during neuronal differentiation of PC12 cells, suggesting that mPRβ is important for neuronal maturity and characteristics.

It was previously reported that progesterone promotes neurite outgrowth^25^. It is thought that the effects of progesterone on neural cells, including previous report, are generally mediated by genomic action via nuclear progesterone receptor^35^. However, we originally showed that mPRβ expression is drastically increased in association with neuronal differentiation, and mPRβ promotes neurite outgrowth through non-genomic effects via the activation of the PI3K-Rac1-MAPK cascade by progesterone. Our data revealed, at least partially, the mechanism underlying progesterone-dependent neurogenesis.

mPRβ has been identified as a putative GPCR^18^. However, our data indicate that mPRβ functions are not related to Gi/o, involved in the inhibition of cAMP production; Gq, involved in the elevation of [Ca^2+^]_i_. Hence, similar to mPRα and mPRγ, mPRβ does not present GPCR characteristics. All mPRs are probably not GPCR, because receptors belonging to the Paqr family, including adipoR1 and adipoR2, present an incomplete GPCR topology. We showed that mPRβ promotes the activation of the MAPK cascade independently of GPCR. AdipoR1 and AdipoR2 promotes AMPK phosphorylation and elevation of [Ca^2+^]_i_ independently of GPCR^36^. However, progesterone promoted AMPK phosphorylation without mPRβ activation. Our data indicate that progesterone sufficiently activates ERK at a concentration of 10 μM compared to the activation of AMPK at a concentration of 100 μM. This difference in the concentration for activation may also explain the promotion of ERK phosphorylation by the membrane progesterone receptor mPRβ and the promotion of AMPK phosphorylation by the other progesterone receptor or a different mechanism. Additionally, progesterone-stimulated mPRβ activation did not exhibit the elevation of [Ca^2+^]i. The signaling of mPRs shows no communality in Paqr family and the detailed intracellular signaling pathway remains unclear.

Thus, mPRβ exerts interesting effects via non G protein signaling as a membrane progesterone receptor. However, PC12 and SH-SY5Y cells are neuroblastoma and not native neural cells. Hence, further studies of mPRβ functions on the subtypes of neurons that express mPRβ in human and mouse primary cultured neuronal cells are lead to verify interspecies commonality and relationship to progesterone-derived physiological functions in nervous system. Additionally, although several reports described how the binding of progesterone to mPRs, including mPRβ, induces biological responses, the exact function of mPRs in progesterone signaling remains obscure. The knockout of mPR genes in mice has not yet been reported. Therefore, the *in vivo* functions of mPRs remain unclear. In the future, mPR gene knockout in mice will provide insights on the intracellular signaling pathways activated by mPRs and on their physiological functions.

In this study, we showed that stimulation of mPRβ by progesterone promotes neurite outgrowth via activation of the MAPK cascade without GPCR signaling. These findings indicate that the binding of progesterone to mPRβ results in non-genomic actions in the CNS. This could represent a central mechanism underlying the unclear effects of progesterone on sex difference-related body homeostasis. Our results may contribute to the development of drugs for treatment of neurological diseases such as ischemic stroke, traumatic brain injury, subarachnoid hemorrhage, and diabetic peripheral neuropathy.

## Materials and Methods

### Animals

C57BL6/J mice were housed under a 12-h light–dark cycle and given regular chow (MF, Oriental Yeast Co, Tokyo, Japan). All experimental procedures involving mice were performed according to protocols approved by the Committee on the Ethics of Animal Experiments of the Tokyo University of Agriculture and Technology. (Permit Number: 28–87).

### RNA extraction and real-time quantitative RT-PCR

Total RNA was extracted using an RNeasy Mini Kit (Qiagen, Chatsworth, CA, USA). cDNA was transcribed from RNA as a template with Moloney murine leukemia virus reverse transcriptase (Invitrogen, Carlsbad, CA, USA). The cDNA was amplified by PCR with Taq DNA polymerase (Nippon Gene, Tokyo, Japan) using primers shown in Supplementary Table S1. The amplified DNA was analyzed by 1.5% agarose gel electrophoresis and the gel was stained with ethidium bromide. Real-time quantitative RT-PCR analyses were performed using DNA Engine Opticon-2 (MJ Research, Waltham, MA, USA) as described previously^37^. For each condition, expression was quantified in duplicate.

### Western blotting

Tissues were homogenized in 0.1 M sodium phosphate buffer, pH 7.4, and centrifuged at 14,000 g for 30 min at 4 °C. PC12 cells were seeded at a density of 1 × 10^5^ cells per well in 24-well plates coated with poly-L-lysine (20 μg/mL). The cells were cultured in DMEM containing NGF (50 ng/mL) and 1% FBS for 24 h, and then in serum-free DMEM for 3 h. The cells were further cultured for 10 min in the presence of progesterone (10 μM; Wako Pure Chemical Industries, Osaka, Japan). Flp-In T-REx HEK293 cells were seeded at a density of 1 × 10^5^ cells per well in 24-well plates. After 24 h, the cells were cultured in DMEM containing 10 μg/mL doxycycline and 10% FBS for 24 h. Cells were further cultured in serum-free DMEM containing doxycycline (10 μg/mL) for 24 h. The cells were further cultured for 10 min in the presence of progesterone (10 μM). Cells were lysed in TNE buffer containing 10 mM Tris-HCl (pH 7.4), 150 mM NaCl, 1 mM EDTA, 1% Nonidet P-40, 50 mM NaF, 2 mM Na3VO4, 10 g/mL aprotinin, and 1% Phosphatase inhibitor cocktail (Nacalai Tesque, Kyoto, Japan). Proteins in the cell lysate were resolved by SDS gel electrophoresis and blotted onto a nitrocellulose membrane. β-Actin, mPRβ, AMPK, ERK1/2, Akt, Rac and its activated forms were detected by western blotting using antibodies. Primary antibodies used were as follows: rabbit antibodies against ERK1/2 (1:1000) (Cell Signaling, Danvers, MA, USA), phosphorylated ERK1/2 (1:1000) (Cell Signaling), AMPKalpha (1:1000), phosphorylated AMPKalpha (1:1000) (Cell Signaling), Akt (1:1000) (Cell Signaling, Danvers, MA, USA), and phosphorylated Akt (1:1000) (Cell Signaling), mPRβ (1:1000) (Bioss, Woburn, MA), mouse antibodies against β-Actin (1:5000) (Wako) and Rac1 (1:1000) (Millipore). The secondary antibody used was a horseradish peroxidase-conjugated Donkey anti-rabbit antibody (1:2000) (GE Healthcare) and horseradish peroxidase-conjugated Sheep anti-mouse antibody (1:5000) (GE Healthcare). Immunoreactive bands were visualized using an enhanced chemiluminescence detection system as described^38^. Image J (National Institutes of Health) was used to quantify the integrated density of each band.

### *In situ* hybridization

For the *in situ* hybridization of sections, mouse embryos and brains were frozen in powdered dry ice, and 16 μm sections were cut using a cryostat and stored at −80 °C until hybridization. ^35^S-labeled mouse antisense *mPRβ* RNA probe was transcribed using T7 RNA polymerase with uridine 5′-α-[^35^S] thiotriphosphate (GE Healthcare, Chicago, IL, USA). The sections were examined by *in situ* hybridization using a labeled probe, followed by exposure to X-ray films (BioMax MR; Kodak, Rochester, NY, USA) for 10 days as described previously^37^. The sections of mouse embryos and brains were counterstained with hematoxylin-eosin.

### Primary culture

Cultured astrocytes were prepared from mouse embryonic cerebral cortex (post-natal day 1) as described previously^39^. Cultured mouse cerebral cortical cells were prepared from mouse embryonic cerebral cortex (E18.5) as described previously^40^. Mouse neural precursor cells were prepared from mouse embryonic cerebral cortex (E13.5) as described previously^41^.

### Culture of PC12 cells, SH-SY5Y, and HEK293 cells

PC12 cells were seeded into DMEM containing 1% penicillin–streptomycin solution (Gibco, Grand Island, NY, USA), 10% HS, and 5% FBS. SH-SY5Y cells were seeded into DMEM containing 1% penicillin–streptomycin solution, and 10% FBS. HEK293 cells were seeded into DMEM containing 10 μg/mL blasticidin S (Funakoshi, Tokyo, Japan), 100 μg/mL hygromycin B (Gibco), and 10% FBS. The cells were incubated at 37 °C in an atmosphere of 5% CO_2_. The cells were further cultured under various conditions.

### Quantification of neurite outgrowth

PC12 cells cells were plated onto 35-mm dishes coated with poly-L-lysine (20 μg/mL; Sigma, St. Louis, MO, USA) at a density of 1 × 10^5^ cells per dish in DMEM supplemented with 10% HS and 5% FBS. After 24 h in culture, the cells were further cultured in DMEM containing NGF (50ng/mL) and 1% FBS for 3 days. SH5Y cells were plated onto 24-well plates coated with poly-L-lysine (20 μg/mL) at a density of 2.5 × 10^4^ cells per well in DMEM supplemented with 10% FBS. After 24 h in culture, the cells were further cultured in DMEM containing NGF (50 ng/mL) and 1% FBS. At least more than 200 cells in each of the dishes were scored. Cells with outgrowths longer than diameter of the cell body were scored positive for neurites. ImageJ (National Institutes of Health, Behesda, MD, USA) was used to measure neurite outgrowth^42^.

### Knockdown of mPRβ expression by siRNA

PC12 cells were transfected with 200 nM of siRNA as shown in Supplementary Table S2 (Bonac corporation, Fukuoka, Japan) by using Lipofectamine 2000 transfection reagent (Invitrogen). For all relative control experiments, cells were exposed to a scrambled non-specific control siRNA from Dharmacon (CAT#ID D-001810-01-05, Dharmacon, Lafayette, CO, USA). The knockdown of mPRβ expression was examined by RT-PCR as described previously^43^. The transfected cells were cultured in DMEM containing 10% HS and 5% FBS for 24 h and then in DMEM containing NGF (50 ng/mL) and 1% FBS.

### [Ca^2+^]_i_ response analysis

Cells were seeded at a density of 1 × 10^5^ cells per well on poly-L-lysine coated 96-well plates, incubated at 37 °C for 24 h, and then incubated in Hanks’ Balanced Salt Solution, pH 7.4, containing calcium assay kit component A (Molecular Devices, Sunnyvale, CA, USA) for 1 h at room temperature. Progesterone used in the Functional Drug Screening System (Hamamatsu Photonics, Shizuoka, Japan) assay was dissolved in Hanks’ Balanced Salt Solution (with 1% EtOH) and prepared in another set of 96-well plates. These plates were set on the Functional Drug Screening System, and mobilization of [Ca^2+^]_i_ was monitored^44^.

### cAMP determination

PC12 cells and HEK293 cells were plated onto 24-well plates and after 24 h in culture, each well was treated with NGF (50 ng/mL) or doxycycline (10 μg/mL) for 24 h. cAMP concentration was determined by enzyme immunoassay (EIA) using cAMP EIA kit (Cayman Chemical, Ann Arbor, Michigan, USA) according to the manufacturer’s protocol. For cAMP determination, the cells were lysed in a 0.1-N HCl solution^45^. We conducted the assays in duplicate.

### Prediction of membrane helices

The amino acid sequence of mouse mPRβ (GenBank Accession numbers: NM_028829) was retrieved from GenBank. The obtained sequence was analyzed by using TMHMM Server v. 2.0 (http://www.cbs.dtu.dk/services/TMHMM/) with default settings.

### Localization analysis

For transfection, HEK293 cells were plated on poly-lysine coated chamber slides (SCS-008, Matsunami, Japan) in DMEM medium containing 10% FBS. HEK293 cells on chamber slide at 80% confluency were transfected with plasmids expressing N-terminal FLAG-tagged mPRβ, C-terminal His-tagged mPRβ, or N-terminal FLAG-tagged GPR41. Briefly, 1 μg of plasmids were added in 50 μL Opti-MEM I medium. Lipofectamine 2000 (2 μL) (Invitrogen) were separately prepared in 50 μL Opti-MEM I medium and incubated for 5 min at room temperature. The two solutions were mixed, and then incubated for 20 min at room temperature. This mixture was added to HEK293 cells and the cells were incubated overnight at 37 °C in a 5% CO_2_ incubator.

The cells were fixed in 4% formaldehyde in PBS for 10 min at room temperature and incubated with 0.1% Triton-X in PBS or PBS alone for 5 min at room temperature. After washing with PBS, the cells were pre-incubated for 1 h in 1% BSA in PBS, and then probed with the Alexa488-conjugated mouse anti-His-tag antibody (MBL, Japan) at a dilution of 1:200 in 1% BSA in PBS or Alexa488-conjugated mouse anti-FLAG antibody (MBL) at a dilution of 1:200 in 1% BSA in PBS for 1 h at room temperature. After washing twice with PBS, the cells were observed using a Zeiss LSM700 confocal microscope.

### Generation of HEK293 cells expressing mouse mPRβ

Flp-In T-REx HEK293 cells were transfected with a mixture of mouse Etag-mPRβ cDNA in pcDNA5/FRT/TO vector and the pOG44 vector using Lipofectamine reagent (Invitrogen). After 48 h, the medium was replaced by medium supplemented with 200 μg/mL hygromycin B to initiate the selection of stably transfected cells. Following the isolation of resistant cells, the expression of mPRβ from the Flp-In locus was induced by treatment with 10 μg/mL doxycycline for 24 h as described previously^46^.

### Immunoprecipitation

The rabbit polyclonal anti-mPRβ antibody (bs-11410R; Bioss Inc) was used to immune-precipitate mPRβ. TrkA was immunoprecipitated using the rabbit polyclonal anti-TrkA antibody (#2505; CST) as described previously^2^. To detect Rac-1 (Rac-1-GTP) in cell lysates, we used a Rac-1/Cdc-42 Activation Assay Kit (17-441, Millipore), using the manufacturer’s instructions. Cells were washed three times with ice-cold PBS and collected by gently scraping using 1 mL of ice-cold MLB Buffer (25 mM HEPES, pH 7.5, 150 mM NaCl, 1% Igepal CA-630, 10 mM MgCl_2_, 1 mM EDTA and 10% glycerol, aprotinin 10 μg/ml).

### Statistical analysis

Values are presented as the mean ± s.e.m. Differences between groups were examined for statistical significance using Student’s t-test (two groups) or one-way analysis of variance followed by Tukey-Kramer’s post hoc test. P-values < 0.05 were considered statistically significant.

## Electronic supplementary material

Supplementary Information
