# Supplementary material for: Membrane progesterone receptor beta (mPRβ/Paqr8) promotes progesterone-dependent neurite outgrowth in PC12 neuronal cells via non-G protein-coupled receptor (GPCR) signaling
Source: Sci Rep. 2017 Jul 12;7:5168. doi: 10.1038/s41598-017-05423-9 (PMC5507890; doi:10.1038/s41598-017-05423-9)
Supplement: Supplementary file 1 — Supplementary Information [file 41598_2017_5423_MOESM1_ESM.pdf]

Membrane progesterone receptor beta (mPRβ/Paqr8) promotes progesterone-dependent neurite outgrowth in PC12 neuronal cells via non-G protein-coupled receptor (GPCR) signaling

Mayu Kasubuchi, Keita Watanabe, Kanako Hirano, Daisuke Inoue, Xuan Li, Kazuya Terasawa, Morichika Konishi, Nobuyuki Itoh, Ikuo Kimura

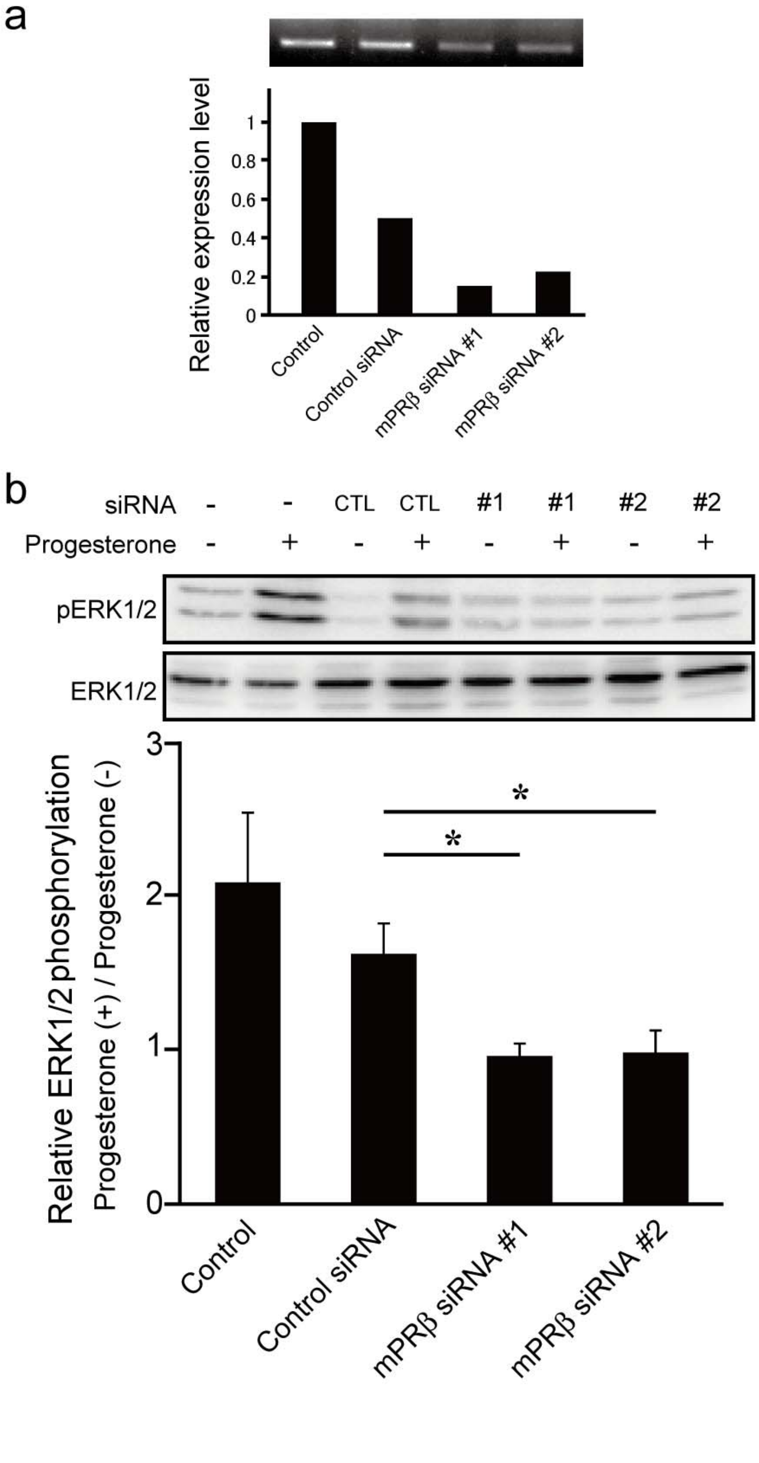

Supplementary Figure 1. mPRβ siRNA significantly suppressed the progesterone-stimulated ERK phosphorylation.

(a) Expression of *mPRβ* mRNA in NGF-induced neuronal PC12 cells. After being treated with Control siRNA or mPRβ siRNA, PC12 cells were cultured for 24 h in DMEM containing 1% FBS, NGF (50 ng/mL). mPRβ cDNA (about 600 base pairs) was detected by 1.5% agarose gel electrophoresis followed by staining with ethidium bromide. Expression of mPRβ was measured by using quantitative RT-PCR. *18S* mRNA expression was used as an internal control. (b) Inhibitory effects of mPRβ siRNA on the phosphorylation of ERK1/2 in NGF-induced neuronal PC12 cells. After being treated with Control siRNA or mPRβ siRNA, cells were cultured for 3 days in DMEM containing 1% FBS, NGF (50 ng/mL) and with or without progesterone (10 μM). ERK1/2 and phosphorylated ERK1/2 in cells were detected by western blotting with specific antibodies. (n = 3). CTL: Control. Results are presented means ± S.E.M. *$p < 0.05$.

Supplementary Table 1. Primers used for PCR.

Primer	Forward	Reverse
mPRα **(mouse)**	5'-CGGCATGGCGATGGCAGTA -3'	5'-ACCCACCCGCTTTCACTTGG -3'
mPRβ **(mouse)**	5'- CACCGCTGTCATGACGACT -3'	5'- CCTCATTTGAAGGACCTCAGG 3'
mPRα **(rat)**	5'- TATGCCATAGAGCCGTCCTG -3'	5'- GCTTATAGATGGCTCCCCGA -3'
mPRβ **(rat)**	5'- GAGCTGTCCCACTACACCTT -3'	5'- GAGAGCGTGCAGATGGAAAG -3'
PR **(rat)**	5'- GCATCCTGTACAAAGCGGAG -3'	5'- GTTATGCTGCCCTTCCATCG -3'
PGRMC1 **(rat)**	5'- TTTTCACGTCGCCTCTCAAC -3'	5'- TTCAGCAGTTTTCCCACGTG -3'
mPRb **(human)**	5'- GCATCCTGTACAAAGCGGAG -3'	5'- GTTATGCTGCCCTTCCATCG -3'

18S **(rat, mouse)**	**5'- ACGCTGAGCCAGTCAGTGTA -3'**	**5'- CTTAGAGGGACAAGTGGCG -3'**
18S **(human)**	**5'-AGATCTGTCAAGTGGTGCCA -3'**	**5'- AAGTACTTCCCGGGTTCCTG-3'**

Supplementary Table 2. siRNA used for RNAi.

siRNA	Sequence
mPRβ #1	**5'- UACCACUUGACAGAUCUU -3'**
mPRβ #2	**5'- UUUCUUAAUCAGUCUGUC -3'**
